# Association Between Postoperative Thrombocytopenia and Outcomes After Coronary Artery Bypass Grafting Surgery

**DOI:** 10.3389/fsurg.2021.747986

**Published:** 2021-09-17

**Authors:** Jinghang Li, Dongmin Yu, Yuanyuan Song, Iokfai Cheang, Xiaowei Wang

**Affiliations:** ^1^Department of Cardiovascular Surgery, The First Affiliated Hospital of Nanjing Medical University, Nanjing, China; ^2^Department of Cardiology, The First Affiliated Hospital of Nanjing Medical University, Nanjing, China

**Keywords:** coronary artery bypass grafting, thrombocytopenia, mortality, MIMIC-III database, perioperation

## Abstract

**Objectives:** The effect of postoperative thrombocytopenia on adverse events among coronary artery bypass graft (CABG) patients remains unclear. This study aims to investigate the association between postoperative thrombocytopenia and perioperative outcomes of CABG.

**Methods:** This is a retrospective study with MIMIC-III (Medical Information Mart for Intensive Care III) database. Adult patients who underwent CABG were included to analyze the impact of thrombocytopenia in patients' outcomes. Postoperative thrombocytopenia was defined as a platelet count <100 × 10^9^/L on the first day after CABG surgery. A multivariable logistic regression analysis was utilized to adjust the effect of thrombocytopenia on outcomes for baseline and covariates, and to determine the association with outcomes.

**Results:** A total of 4,915 patients were included, and postoperative thrombocytopenia occurred in 696 (14.2%) patients. Postoperative thrombocytopenia was not associated with increased 28-day mortality (OR 0.75; 95% CI 0.33–1.72; *P* = 0.496) or in-hospital mortality (OR 0.75; 95% CI 0.34–1.63; *P* = 0.463) after adjusting for confounders. Regarding the secondary outcomes, it was associated with a higher risk of a prolonged stay in the intensive care unit (OR 1.53; 95% CI 1.18–1.97; *P* = 0.001), prolonged hospital stays (OR 1.58; 95% CI 1.21–2.06; *P* = 0.001), prolonged mechanical ventilation time (OR 1.67; 95% CI 1.14–2.44; *P* = 0.009), and a trend toward increased occurrence of massive bleeding (OR 1.41; 95% CI 1.00–2.01; *P* = 0.054). There was no significant association between an increased risk of prolonged vasopressor use and the continuous renal replacement therapy rate.

**Conclusions:** Postoperative thrombocytopenia was associated with prolonged ICU and hospital stays but not with increased perioperative mortality among CABG patients.

## Introduction

The causes of thrombocytopenia in cardiac surgery are multifactorial and complicated. Studies suggested inflammatory response induced by cardiopulmonary bypass (CPB) as a major factor. The activation of inflammatory cytokines (such as TNF-α, IL-8, IL-10, IL-6, and IL-1β) (1, 2) could induce systemic autoimmune platelet clearance (3, 4). The other risk factors included hemodilution, platelet consumption and destruction, heparin-induced thrombocytopenia (HITP), drug-induced thrombocytopenia (DITP), etc. (5).

A low platelet count is one common indicator of organ dysfunction in intensive care unit (ICU), which often relates to the severity and mortality of critical diseases (5). Previous studies showed that preoperative thrombocytopenia was associated with both severe bleeding and worse outcomes in both cardiac and non-cardiac surgery (6, 7). A recent study of coronary artery bypass grafting (CABG) patients with preoperative thrombocytopenia showed a significant association with severe bleeding and increased 30-day and 1-year mortality (8).

Furthermore, CABG patients receiving antiplatelet therapy exposed to an increased risk of perioperative bleeding and reduced platelet count. As platelet count could reflect a systemic organ function, thrombocytopenia in CABG patients not only relates to bleeding events but also predicts other postoperative adverse events, including acute kidney injury, stroke, and acute myocardial infarction (9–11). Therefore, comprehensively evaluating the impact of perioperative thrombocytopenia in CABG patients is of great importance.

In this study, we characterized the incidence of postoperative thrombocytopenia on the first day after CABG surgery in the MIMIC-III database. Our study aims to explore whether thrombocytopenia in CABG patients has a relationship with an increased risk for postoperative mortality and other adverse events.

## Methods

### Sources of Data

The current large single-center retrospective cohort study used publicly available data from the Medical Information Mart for Intensive Care (MIMIC) III database conducted by Beth Israel Deaconess Medical Center (BIDMC, Boston, Massachusetts). Study was in accordance with the MIMIC-III guidelines which approved by the institutional review boards. Further details on MIMIC-III ethics are available from its original publication (12). Database contains data from 46,520 ICU patients admitted to the BIDMC from 2001 to 2012, including diagnosis, demographics, procedures, vital signs, laboratory examination results, input and output information, medications, and other clinical variables.

### Data Collection and Definitions

All patients undergoing isolated CABG were enrolled in this analysis. Exclusion criteria were age <18 years old; lack of platelet counts record within 24 h ICU admission, and >5% missing variables. The patients' baseline characteristics, comorbidities, vital signs, mean arterial pressure (MAP), laboratory results, use of vasopressors, chest drainage volume, continuous renal replacement therapy (CRRT), mechanical ventilation time, etc. were collected. The sequential organ failure assessment (SOFA) score at admission to the ICU was calculated. Postoperative thrombocytopenia was defined as a platelet count <100 × 10^9^/L on the first day after CABG surgery (13).

The primary endpoints were 28-day mortality from the date of ICU admission and in-hospital mortality. Patient mortality information for discharged patients was gathered from the US Social Security Death Index. The secondary outcomes were length of ICU stay, length of hospital stay, mechanical ventilation time, vasopressor use time, chest drainage volume, and CRRT rate.

### Statistical Methods

Continuous variables with non-normal distributions in the present study are presented as the median ± interquartile range. Categorical variables are presented as numbers and percentages. Comparison analyses were conducted by using the Mann-Whitney *U*-test for continuous variables and the χ^2^ test or Fisher exact test for categorical variables.

Univariable logistic regression analysis was conducted to estimate the relationships between postoperative thrombocytopenia and all outcomes. Then, multivariable logistic regression analysis was performed to adjust for the following covariates: comorbidities (hypertension, congestive heart failure, diabetes, chronic pulmonary, renal failure), age, sex, body mass index (BMI), elective surgery, laboratory tests [white blood cell count (WBC), hemoglobin, lactate, activated partial thromboplastin time (APTT), prothrombin time (PT), creatinine], and vital signs [heart rate, mean arterial pressure, respiratory rate, temperature]. To facilitate logistic regression analysis, all continuous outcome variables were transformed into categorical variables according to the 75% interquartile range or clinical definition.

Statistical analysis was performed using STATA version 15 (STATA Corp LLC, College Station, TX, USA) and SPSS version 26.0 (IBM, Armonk, NY). *P* < 0.05 was considered statistically significant.

## Results

### Clinical Characteristics of the Study Population

**Figure 1:**
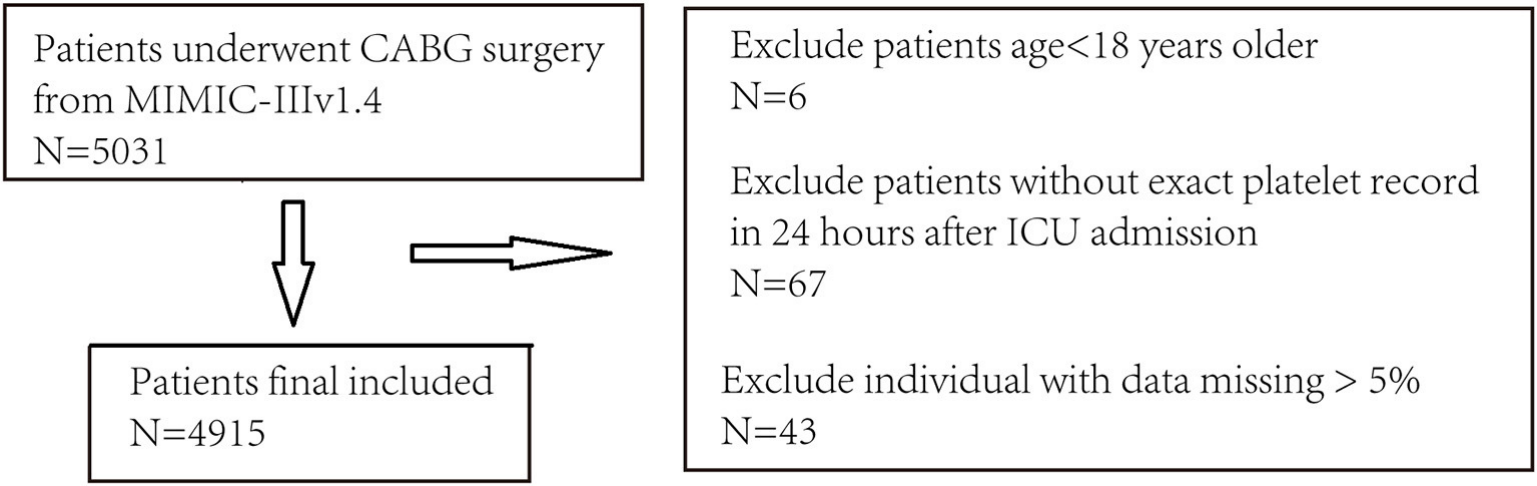
The procedure of data selection and exclusion.

A total of 4,915 CABG patients [median age 68.5 (60.5–76.3) year-old] were included. Median platelet count was 146 [115–184] × 10^9^/L. The data selection procedure is presented in Figure 1.

There were 696 (14.2%) patients had developed postoperative thrombocytopenia. The platelet count was significantly lower in the postoperative thrombocytopenia group (84 [71–92] vs. 155 [128–191] × 10^9^/L, *P* < 0.001). The baseline characteristics between groups with and without postoperative thrombocytopenia are presented in Table 1. The postoperative thrombocytopenia group was older (median age 73.6 vs. 67.6 years old); had fewer males (68.5 vs. 75.4%); had a lower BMI, WBC, hemoglobin level, respiratory rate, and diabetes rate; and had a higher congestive heart failure rate, lactate level, APTT, PT and SOFA score. There were no significant differences in heart rate, MAP, comorbidity of hypertension, chronic pulmonary disease, renal failure, and elective surgery between groups.

**Table 1:** Baseline and clinical characteristics of the study population.

**Variables**	**Thrombocytopenia *N* = 696**	**Non-thrombocytopenia *N* = 4,219**	***P***
Age, years	73.6 (65.9, 80.0)	67.6 (59.6, 75.4)	<0.001
Gender, male, *n* (%)	477 (68.5%)	3,180 (75.4%)	<0.001
BMI	26.5 (23.6, 29.9)	28.2 (25.1, 31.9)	<0.001
**Comorbidities**
Hypertension, *n* (%)	515 (74.0%)	3,174 (75.2%)	0.479
Congestive heart failure, *n* (%)	223 (32.0%)	1,045 (24.8%)	<0.001
Diabetes, *n* (%)	220 (31.6%)	1,667 (39.5%)	<0.001
Chronic pulmonary disease, *n* (%)	88 (12.6%)	625 (14.8%)	0.146
Renal failure, *n* (%)	75 (10.8%)	419 (9.9%)	0.496
**Laboratory tests**
WBC, 10^9^/L	12.1 (9.4, 15.0)	14.0 (11.1, 17.5)	<0.001
Platelet, 10^9^/L	84 (71, 92)	155 (128, 191)	<0.001
Hemoglobin, g/L	76 (67, 87)	86 (75, 97)	<0.001
Lactate, mmol/L	3.2 (2.2, 4.4)	2.4 (1.9, 3.2)	<0.001
Creatinine, mg/dl	1.0 (0.8, 1.2)	0.9 (0.8, 1.2)	0.012
APTT, seconds	47.1 (37.0, 65.2)	37.0 (31.6, 48.2)	<0.001
PT, seconds	16.7 (15.5, 18.4)	15.2 (14.3, 16.3)	<0.001
**Vital signs**
Heart rate, bpm	84.9 (79.2, 90.0)	84.7 (78.9, 90.9)	0.966
MAP, mmHg	74.3 (70.6, 78.2)	74.2 (70.6, 78.4)	0.633
Respiratory rate, bpm	16.1 (14.6, 18.1)	16.9 (15.3, 18.8)	<0.001
Temperature, °C	37.7 (37.3, 38.1)	37.6 (37.2, 38.1)	0.04
Elective surgery, *n* (%)	263 (37.8%)	1,509 (35.8%)	0.307
SOFA score	6 (5,8)	4 (3,6)	<0.001
SOFA score>2, *n* (%)	685 (98.4%)	3,298 (78.2%)	<0.001

*Values are expressed as median (interquartile range), or n (%)*.

*BMI, body mass index; WBC, white blood cell; APTT, activated partial thromboplastin time; PT, prothrombin time; MAP, mean arterial pressure; SOFA, sequential organ failure assessment*.

*In bold are statistically significant values*.

### Related Risk Factors for Postoperative Thrombocytopenia

The multivariable logistic regression analysis showed that age (OR 1.042; 95% CI 1.028–1.055), lactate (OR 1.221; 95% CI 1.143–1.306), APTT (OR 1.006; 95% CI 1.001–1.011), and PT (OR 1.083; 95% CI 1.041–1.126) were associated with a significantly higher risk of postoperative thrombocytopenia; BMI (OR 0.963; 95% CI 0.939–0.988), WBC (OR 0.892; 95% CI 0.866–0.918), and hemoglobin (OR 0.831; 95% CI 0.759–0.910) were associated with a significantly lower risk of postoperative thrombocytopenia (Table 2).

**Table 2:** The risk factors of postoperative thrombocytopenia in multivariable logistic regression analysis.

**Variables**	**OR**	**95%CI**	**P-value**
Age	1.042	1.028, 1.055	** <0.001**
BMI	0.963	0.939, 0.988	**0.004**
WBC	0.892	0.866, 0.918	** <0.001**
Hemoglobin	0.831	0.759, 0.910	** <0.001**
Lactate	1.221	1.143, 1.306	** <0.001**
APTT	1.006	1.001, 1.011	**0.011**
PT	1.083	1.041, 1.126	** <0.001**

*Estimates are odds ratios with 95% confidence interval*.

*BMI, body mass index; WBC, white blood cell; APTT, activated partial thromboplastin time; PT, prothrombin time*.

*In bold are statistically significant values*.

### Impact of Postoperative Thrombocytopenia on Primary and Secondary Outcomes

Univariate logistic regression analysis showed both the primary outcomes, 28-day mortality (3.0 vs. 1.2%, *P* = 0.001) and in-hospital mortality (3.4 vs. 1.4%, *P* = 0.001), were significantly increased in postoperative thrombocytopenia patients. While the secondary outcomes, length of ICU stay (2.8 [1.3, 4.6] vs. 2.2 [1.2, 3.9], *P* < 0.001), length of hospital stay (8.3 [6.2, 12.7] vs. 7.6 [5.4, 11.0], *P* < 0.001), length of ventilation time (12.0 [4.7, 21.7] vs. 5.9 [3.5, 15.6], *P* < 0.001), length of vasopressor usage (14.2 [3.0, 38.4] vs. 12.0 [1.3, 29.9], *P* < 0.001), and chest drainage volume (1,062.5 [762.5, 1,648.8] vs. 800 [535, 1,150], *P* < 0.001), were also significantly increased in postoperative thrombocytopenia patients (Table 3).

**Table 3:** Clinical outcomes between study cohorts.

**Outcomes**	**Thrombocytopenia *N* = 696**	**Non-thrombocytopenia *N* = 4,219**	***P*-value**
**Primary outcomes**			
28-day mortality, *n* (%)	21 (3.0%)	50 (1.2%)	**0.001**
In-hospital mortality, *n* (%)	24 (3.4%)	61 (1.4%)	**0.001**
**Secondary outcomes**			
Length of ICU stay, days	2.8 (1.3, 4.6)	2.2 (1.2, 3.9)	** <0.001**
Length of hospital stay, days	8.3 (6.2, 12.7)	7.6 (5.4, 11.0)	** <0.001**
Length of ventilation time, hours	12.0 (4.7, 21.7)	5.9 (3.5, 15.6)	** <0.001**
Length of vasopressor time, hours	14.2 (3.0, 38.4)	12.0 (1.3, 29.9)	** <0.001**
Pericardial and chest drainage, ml	1,062 (762, 1,648)	800 (535, 1,150)	** <0.001**
CRRT first day	11 (1.6%)	62 (1.5%)	0.738

*Values are expressed as median (interquartile range), or n (%)*.

*CRRT, continuous renal replacement therapy*.

*In bold are statistically significant values*.

Further multivariate logistic regression analysis showed that 28-day mortality (OR 0.75; 95% CI 0.33–1.72; *P* = 0.495) and in-hospital mortality (OR 0.75; 95% CI 0.34–1.63; *P* = 0.463) did not show significance increased risk in patients with postoperative thrombocytopenia after adjusting with the confounders. For the secondary outcomes, postoperative thrombocytopenia was associated with a higher risk of an ICU stay ≥3 days (OR 1.53; 95% CI 1.18–1.97; *P* = 0.001), hospital stay ≥10 days (OR 1.58; 95% CI 1.21–2.06; *P* = 0.001), mechanical ventilation time ≥48 h (OR 1.67; 95% CI 1.14–2.44; *P* = 0.009), and a trend toward an increasing risk with chest drainage volume ≥1,000 ml (OR 1.41; 95% CI 1.00–2.01; *P* = 0.054) (Table 4).

**Table 4:** The associations between postoperative thrombocytopenia and clinical outcomes.

	**Univariate analysis**		**Multivariate analysis**	
	**OR (95%CI)**	***P***	**OR (95%CI)**	***P***
**First outcomes**
28-day mortality	2.59 (1.55, 4.35)	** <0.001**	0.75 (0.33, 1.72)	0.496
In-hospital mortality	2.43 (1.51, 3.93)	** <0.001**	0.75 (0.34, 1.63)	0.463
**Secondary outcomes**				
Length of ICU stay ≥3 days	1.50 (1.27, 1.76)	** <0.001**	**1.53 (1.18, 1.97)**	**0.001**
Length of hospital stay ≥10 days	1.35 (1.15, 1.60)	** <0.001**	**1.58 (1.21, 2.06)**	**0.001**
Length of ventilation time ≥48 h	1.99 (1.57, 2.52)	** <0.001**	**1.67 (1.14, 2.44)**	**0.009**
Length of vasopressor time ≥24 h	1.23 (1.04, 1.46)	**0.018**	1.11 (0.84, 1.45)	0.464
Pericardial and chest drainage ≥1,000 ml	2.39 (1.75, 3.26)	** <0.001**	**1.41 (1.00, 2.01)**	**0.054**
CRRT first day	1.08 (0.56, 2.06)	0.823	1.13 (0.23, 3.82)	0.846

*Estimates are odds ratios with 95% confidence interval*.

*CRRT, continuous renal replacement therapy*.

*In bold are statistically significant values*.

## Discussion

In this retrospective study, postoperative thrombocytopenia (14.2%) often occurred in the first day after CABG surgery among of 4,915 patients. The primary outcome −28-day mortality and in-hospital mortality were significantly higher in the patients with postoperative thrombocytopenia. In further multivariable logistic regression analysis, although postoperative thrombocytopenia had a significantly increased risk of prolonged ICU stay, prolonged hospital stays, prolonged mechanical ventilation time, increased risk of massive bleeding, results did not find significant difference after adjusting with cofounders in the primary outcome.

Platelets play an important role in the pathogenesis of multiple organ dysfunction, including acute respiratory distress syndrome (ARDS), acute myocardial infarction (AMI), stroke, massive bleeding, and acute kidney injury (AKI), major bleeding, and could act as a powerful prognostic indicator in critical patients (8, 10, 14–16). Therefore, hypothesis suggested that decreased platelet counts might also associated with an inflammatory end-organ injury and coagulation disorders in cardiac surgery.

However, the role of thrombocytopenia especially in postoperative CABG patients remained controversial. A retrospective study of 4,217 adult CABG surgery patients showed that postoperative thrombocytopenia (platelet count ≤74 × 10^9^/L) was independently associated with an increased risk of 30-day mortality (10). Study of 7,189 CABG surgery patients, preoperative thrombocytopenia (platelet count ≤150 × 10^9^/L) was also associated with an increased risk of both in-hospital/30-day mortality and 1-year mortality (8). On the contrary, the ASCERT study, which included 348,341 CABG patients, did not find platelet count as a predictor of both short-term and long-term mortality after CABG surgery (17). Our results are similar to ASCERT study, thrombocytopenia after CABG surgery did not show significance in the increased risk of short-term mortality.

Platelet as the key component in the process of hemostasis and thrombosis, thrombocytopenia is an important risk factor for postoperative bleeding in most studies of cardiac and non-cardiac surgery (18). Study reported that reduced platelet count was associated with increased postoperative bleeding after cardiac surgery (19); while prospective study of 323 cardiac surgery patients also demonstrated an association of thrombocytopenia with excessive bleeding, and that preoperative platelet count was an independent risk factor for excessive bleeding after cardiac surgery (20). Therefore, the thrombocytopenia cohort had a higher pericardial/chest drainage volume and a lower hemoglobin level, which consisted in our multivariate model. However, platelet count only reflects partial function of the platelet itself and the coagulation function (21). While thrombocytopenia does associate with the risk of adverse outcomes, proper perioperative management might significantly decrease the impact in mortality, especially for CABG patients which usually need to receive anti-platelet management (22).

In the inflammatory aspects, cardiac surgery has a higher risk of acute lung injury (ALI) and/or acute respiratory distress syndrome (ARDS) than non-cardiac surgery and often needs longer ventilation support. The inflammatory response associated with cardiac surgery, blood transfusion and microthrombogenesis may contribute to ALI or ARDS after cardiac surgery. Previous studies have revealed that platelets also contribute to the pathophysiological process of ALI (23). Platelets play a critical role in protecting the integrity of the basal alveolar-capillary barrier and regulating alveolar capillary permeability. When platelet counts are severely decreased, the function of the endothelial barrier is often impaired, leading to increased microvessel permeability, alveolar neutrophil infiltration, and lung injury (24). In the present study, the CABG patients with thrombocytopenia had a significantly prolonged mechanical ventilation time (12 vs. 5.9 h) and a 1.67-fold risk of mechanical ventilation time ≥48 h in the multivariate analysis.

Since postoperative thrombocytopenia count may be caused by inflammation associated with CPB, hemodilution, platelet consumption and destruction, the decrease in postoperative platelet contributes to a systemic adverse mechanism and may exert negative effects by increasing the risk of bleeding, blood transfusion, ALI, poor organ perfusion, and systemic inflammatory response. Although the direct mechanism of postoperative thrombocytopenia on these adverse events has not been well-studied. Our results provided additional evidence regarding platelet in the prognosis of CABG patients, and also established a relationship between thrombocytopenia and other major adverse events. The findings of our study add to the accumulating evidence that postoperative thrombocytopenia is an important risk factor in CABG patients.

## Limitations

This study has several limitations. First, this was a retrospective study based on a public database of the MIMIC III, several potential risk factors and covariates were not available for analysis. For example, the perioperative antiplatelet drug use, types of CABG procedures (off-pump/on-pump), perioperative blood transfusion, etc. are lack in our cohort, which might generate certain bias in our results. Second, this is a single-center study, and the quality of perioperative management and surgical practices may differ from other institutions. Finally, we only studied the impact of the platelet count on CABG surgery, and the influence of platelet function was not considered in this study. Platelet function abnormalities may be a more powerful influencing factor for advent events. Studies with multi-center large cohort, integral covariates analysis, and platelet function are needed to further explore the effects and mechanisms.

## Conclusion

The results of this retrospective study showed that CABG patients with postoperative thrombocytopenia had a significantly increased risk of a prolonged ICU stay, prolonged hospital stay, prolonged mechanical ventilation time and massive bleeding, but the risk of 28-day and in-hospital death were not significantly increased. Further studies using data from other medical centers will be needed to provide further validation of these findings, and the potential mechanism of postoperative thrombocytopenia and its influence on adverse outcomes should be investigated.

## Data Availability Statement

The original contributions presented in the study are included in the article/supplementary material, further inquiries can be directed to the corresponding author/s.

## Ethics Statement

Ethical review and approval was not required for the study on human participants in accordance with the local legislation and institutional requirements. Written informed consent for participation was not required for this study in accordance with the national legislation and the institutional requirements.

## Author Contributions

JL designed the study and coordinated the study. XW provided administrative support. DY and YS gave provision of study materials or patients. JL and IC collected and assembled the data. IC analyzed and interpreted the data. All authors were participated in the manuscript writing and read and approved the final manuscript.

## Conflict of Interest

The authors declare that the research was conducted in the absence of any commercial or financial relationships that could be construed as a potential conflict of interest.

## Publisher's Note

All claims expressed in this article are solely those of the authors and do not necessarily represent those of their affiliated organizations, or those of the publisher, the editors and the reviewers. Any product that may be evaluated in this article, or claim that may be made by its manufacturer, is not guaranteed or endorsed by the publisher.

## Abbreviations

CABG: coronary artery bypass graft
BMI: body mass index
WBC: white blood cell
APTT: activated partial thromboplastin time
PT: prothrombin time
MAP: mean arterial pressure
SOFA: sequential organ failure assessment
CRRT: continuous renal replacement therapy
ARDS: acute respiratory distress syndrome
AMI: acute myocardial infarction
AKI: acute renal failure
ALI: acute lung injury.
